# Trends in Termination of Pregnancy for Foetal Urological Abnormalities in England and Wales: a Cross-Sectional Study

**DOI:** 10.1007/s43032-022-01094-8

**Published:** 2022-09-29

**Authors:** Megan Jeffery, Sarah Tai-MacArthur, Panicos Shangaris, Martin Duggan, Julia Spencer, Srividhya Sankaran

**Affiliations:** 1 GKT School of Biomedical Sciences, King’s College London, Guy’s Campus, Great Maze Pond, London, UK; 2 School of Bioscience Education, King’s College London, Guy’s Campus, Great Maze Pond, London, UK; 3 School of Life Course and Population Sciences, Faculty of Life Sciences and Medicine King’s College London, 10th Floor North Wing St Thomas’ Hospital, London, SE1 7EH UK; 4 Department of Women and Children, Guy’s and St Thomas’ NHS Foundation Trust, London, UK; 5 Peter Gorer Department of Immunobiology, School of Immunology and Microbial Sciences, Faculty of Life Sciences and Medicine, King’s College London, London, UK; 6 Department of Health and Social Care, London, UK

## Abstract

The detection of developmental abnormalities in the foetus is considered an essential component of antenatal screening. Among the most frequently identified sonographically, and possibly one of the easiest recognised, are those affecting the urinary tract, with an incidence of 1–4 in 1000 pregnancies. As such, foetal urological abnormalities represent up to 30% of all prenatally diagnosed congenital anomalies. We analysed information recorded on the Health and Social Act 4 (HSA4) forms submitted to the Department of Health and Social Care (DHSC) for 2015 to 2019. There were 915 cases of termination of pregnancy for foetal urological anomaly between 2015 and 2019 in England and Wales, representing 0.09% of total abortions. There has been a steady increase in cases, from 186 in 2015 to 222 in 2018, followed by a more recent decline in 2019 to 172. All 915 cases were justified under Ground E of The Abortion Act 1967. Most terminations of pregnancy for foetal urological anomaly were carried out at 20 weeks gestation. Isolated urinary tract single diagnoses were the commonest, with megacystis being the most prevalent, followed by bilateral renal agenesis and bilateral cystic kidneys. Nearly a third of cases (32.2%) were performed in women aged 30–34 years, and almost 4/5 of women (78.7%) were of White ethnicity. Foetal urological abnormality is a complex issue affecting a significant minority of pregnant women. When severe abnormalities are detected by prenatal diagnosis, most women choose to terminate the pregnancy.

## Introduction 

The detection of developmental abnormalities in the foetus is considered an essential component of antenatal screening. Among the most frequently identified are those affecting the urinary tract. Congenital foetal anomalies of the kidney and urinary tract (CAKUT) account for 20–30% of all ultrasound-detected anomalies and complicate between 3 and 4% of pregnancies globally [1, 2]. The term CAKUT encompasses a diverse range of structural and functional anomalies at the level of the kidney parenchyma, collecting system, bladder, and/or urethra. The clinical outcomes of each malformation are similarly diverse, ranging from asymptomatic to life-threatening.

Hydronephrosis is the most prevalent and spontaneously resolves in more than 50% of cases [1]. However, the outcome of some urological anomalies are life-limiting and ultimately fatal. The commonest malformations associated with high mortality include bilateral renal agenesis (BRA), autosomal recessive polycystic renal disease (ARPKD), bilateral multicystic dysplastic kidney, and megacystis [2]. Compounding the effect of the original anomaly, diseases of the urinary tract often also impact foetal lung maturation as oligohydramnios/anhydramnios may lead to pulmonary hypoplasia through reduction of intrathoracic cavity size [3]. Ryckeawart et al. suggest that in cases of CAKUT, about 1/3 of foetuses have associated extrarenal malformations, and 10% have abnormal karyotypes [4]. These anomalies, their sequelae, and potential association with syndromes are associated with a high rate of prenatal or neonatal mortality. 19.6% of pregnancies in which a congenital anomaly in the foetal urinary tract has been identified fail to produce a living infant [5].

Advances in prenatal diagnostic technology and screening practice have allowed increasing numbers of such foetal anomalies to be detected. Two large-scale ultrasound screening studies from the 1990s found that of the 954 urological abnormalities detected, almost 90% were detected in the antenatal period (4). This high level of sensitivity for establishing urological malformations before birth and the high degree of reliability for predicting postnatal outcomes are seen universally in research (5). Antenatal identification of urological abnormalities is essential to provide options to prospective parents regarding appropriate pregnancy management as many of these abnormalities considerably affect perinatal morbidity and mortality.

**Fig. 1:**
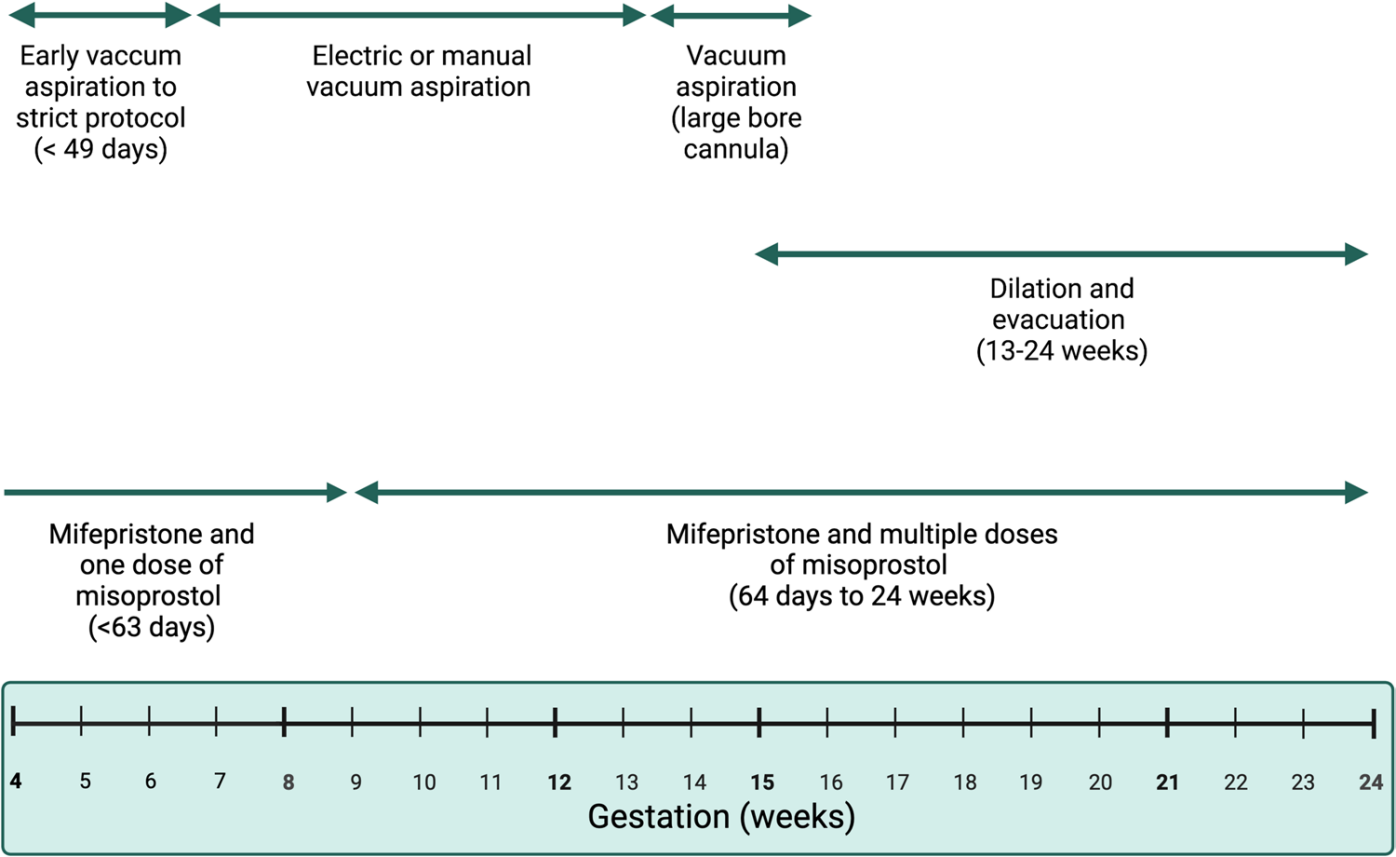
Summary of termination methods appropriate for use in abortion services in the UK by gestational age in weeks, adapted from Kumar et al. [25]

The Abortion Act 1967 legalised abortion in the UK, up to 28 weeks under certain grounds and past this gestational limit only in exceptional medical circumstances. Before the Abortion Act was passed, abortion was illegal in England and Wales under the Offences Against the Person Act 1861. Section 37 of the Human Fertilisation and Embryology Act 1990 amended the Abortion Act. It lowered the 28-week limit to the current 24 weeks and allowed abortions under some grounds at any gestational age which would not be offences under the Infant Life (Preservation) Act 1929. Termination of pregnancy for foetal anomaly (TOPFA) is allowed under Ground E of the Abortion Act, which stipulates there is no gestational age limit if there is ‘ a substantial risk that if the child was born, it would suffer from such physical or mental abnormalities as to be seriously handicapped’. European data suggest the prevalence of urinary anomalies between 2011 and 2018 was 35.43 per 10,000 births, and 4.85 per 10,000 were terminated. This is comparably less than other congenital abnormalities, such as nervous system anomalies, where termination per 10,000 births was 13.79 [6]. However, UK studies suggest that the rate of prenatal diagnosis of CAKUT increases in tandem with TOP associated with CAKUT, suggesting TOPFA for urological anomaly is becoming increasingly common [5]. Different procedures can be used to terminate the pregnancy, depending on the gestational age and other circumstances relating to the individual woman (Fig. 1).

## Aims and Objectives

This study aims to analyse the trends in the termination of pregnancies for foetal urological abnormalities in England and Wales from 2015 to 2019. Other variables, including maternal characteristics and type of termination procedure, were also examined to determine their association with a urological malformation diagnosis.

## Materials and Methods

This is a retrospective observational study, in collaboration with King’s College London and the Department of Health and Social Care (DHSC), looking at the trends in termination of pregnancy for foetal urological abnormalities in England and Wales between 2015 and 2019. The DHSC provided data for analysis of trends, including individual diagnosis, legal grounds, gestation, and maternal characteristics.

### Study Population

Data was collected from the Health and Social Act 4 (HSA4) forms submitted by clinics and hospitals to the DHSC. The DHSC collated aggregated data on abortion statistics relating to CAKUT performed in residents and non-residents in England and Wales between 2015 and 2019. All women, including residents and non-residents, who underwent a termination for a foetal urological abnormality within this period were included. No raw patient-level data was used. A decision tool completed on the NHS Health Research Authority website confirmed that no approval was needed from the Research Ethics Committee. The data was then analysed to identify trends.

## Results

### Prevalence

Between 2015 and 2019, there were 993,767 terminations recorded in England and Wales.

**Fig. 2:**
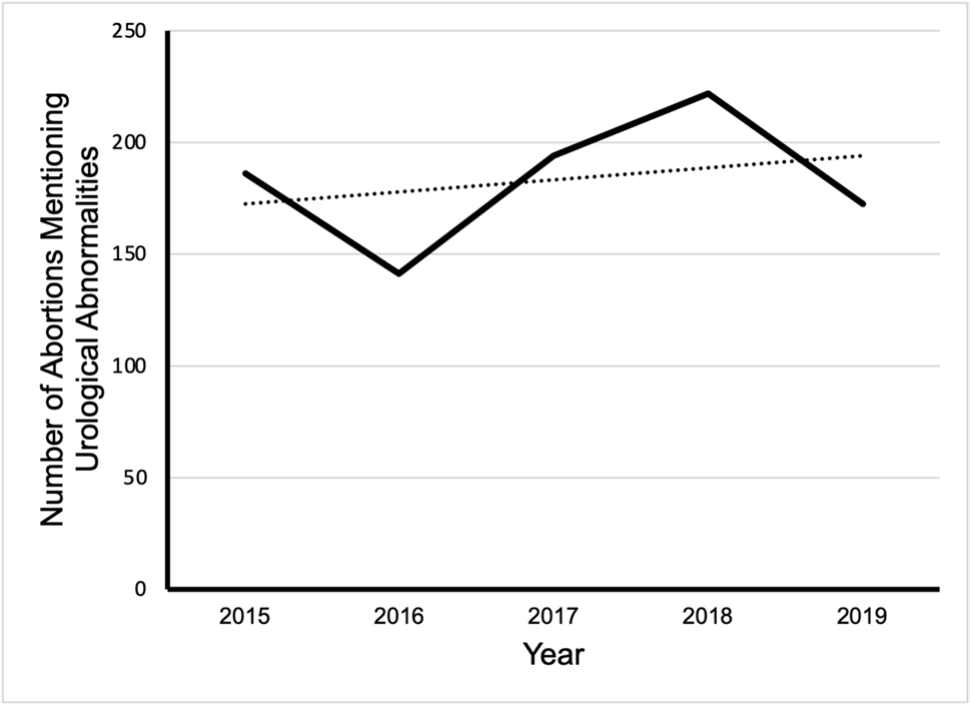
Graph shows the trend over time of the number of abortions performed that mention urological abnormality in residents and non-residents in England and Wales between 2015 and 2019. Data from the DHSC

Within this time frame, 915 terminations mentioned urological abnormalities using the ICD10 codes in the inclusion criteria, representing only 0.09% of the total number of abortions within our study period. Overall, the number of abortions for urological abnormality has remained similar over the 5 years. The total number of cases has decreased by 7.5%, from 186 cases in 2015 and 172 cases in 2019 (Fig. 2). All 915 cases over the 5 years were categorised as Ground E terminations (alone or with Grounds A, B, C, or D).

### Individual Diagnosis

**Fig. 3:**
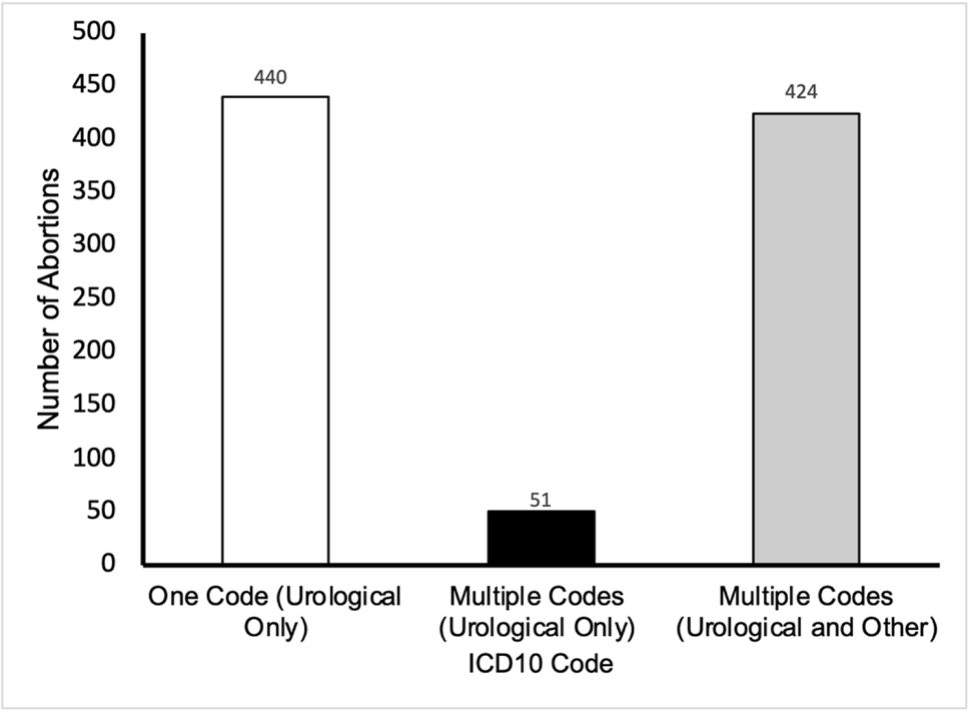
Graph shows the number of abortions where there was an isolated urological anomaly, multiple urological anomalies, and multiple anomalies of both urological and extra-urinary origin in residents and non-residents in England and Wales between 2015 and 2019. Data from the DHSC

**Fig. 4:**
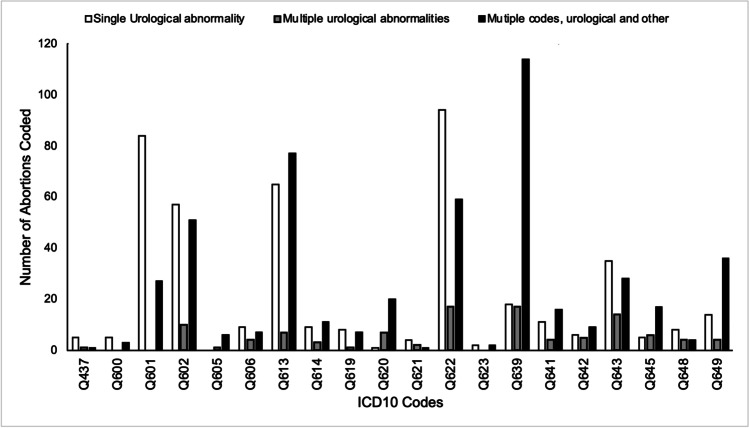
Graph shows the number of times each ICD10 code is recorded for isolated cases, cases with multiple urinary anomalies and issues with urinary and extra-urinary anomalies in residents and non-residents in England and Wales between 2015 and 2019. Data from the DHSC

Of the 915 terminations for foetal urological anomaly, 440 were isolated cases (48.0%) where only a single ICD10 code was recorded (Fig. 3). Within these isolated codes, megacystis (Q622), was the most commonly reported code with a prevalence rate of 21.4%, followed by Q601 bilateral renal agenesis (19.1%), and Q613 multicystic kidney (14.8%) (Fig. 4).

Furthermore, 424 cases (46.3%) had multiple diagnoses including urinary and extra-urinary anomalies, and the smallest proportion, 5.6%, of cases (51) had multiple diagnoses limited to the urinary tract. Renal failure (Q639) was the most frequently reported condition where a further urological diagnosis was made, in addition to megacystis (Fig. 4) which appeared to be most often associated with Q620 (hydronephrosis) and Q643 (bladder outflow problem). Moreover, of those cases where an extra-urinary anomaly was noted, congenital renal failure (Q639) accounted for over a quarter (26.9%) of cases (Fig. 4), followed by the polycystic and multicystic kidney (Q613), which accounted for 18.2% of cases; the extra-urinary anomaly with the highest prevalence was P012 (oligohydramnios), present in almost half (44.5%) of cases with multiple diagnoses not limited to the urinary tract.

### Gestation

**Fig. 5:**
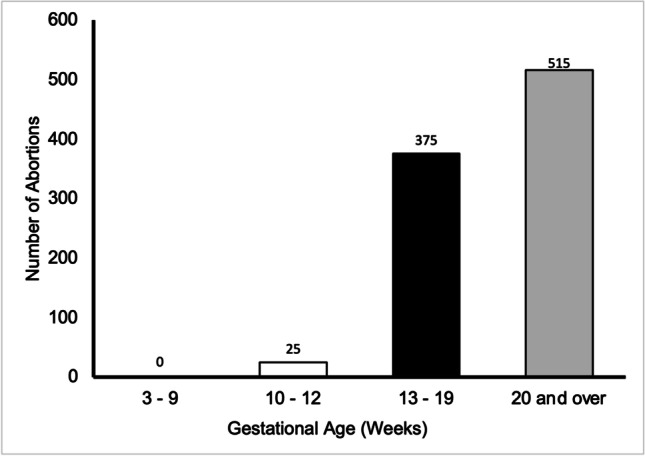
Graph shows the prevalence of abortion for urological abnormality by gestational age in weeks in residents and non-residents in England and Wales between 2015 and 2019. Data from the DHSC

Terminations in England and Wales most commonly occur during the first trimester, with 82% of terminations in 2019 performed under 10 weeks of gestation. The most common gestation to undergo termination for urological abnormality was at 20 weeks or over (Fig. 5), where 56.3% were carried out during this time—515 cases. Of the cases, 41.0% were performed between 13 and 19 weeks of gestation, and only 25 cases occurred between 10 and 12 weeks of gestation, accounting for merely 2.7%. No terminations occurred preceding 10 weeks gestation.

### Method

**Fig. 6:**
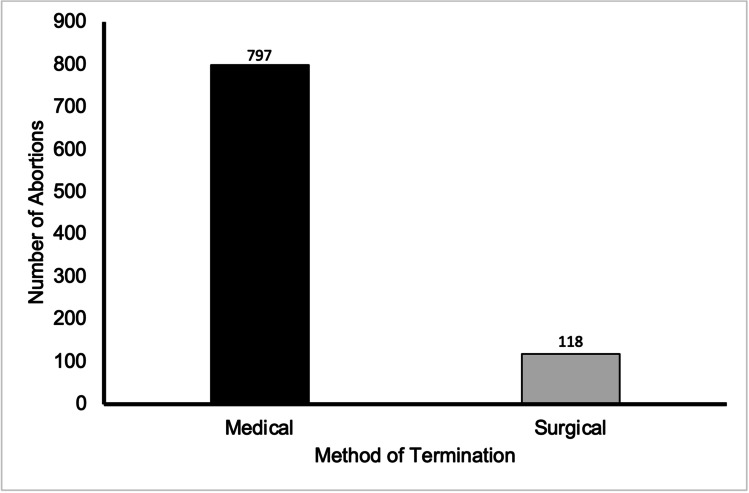
Graph to show the prevalence of abortion for urological abnormality by the method in residents and non-residents in England and Wales between 2015 and 2019. Data from the DHSC

Of all abortions performed for urological anomaly within the time period, 87.1% were terminated medically, and the remaining 12.9% were performed via either of the surgical methods. For all Ground E cases between 2015 and 2019, 73.5% (11,894 cases) used medical methods of termination and 26.5% were terminated using surgical means (Fig. 6).

### Maternal Age

For terminations on the grounds of urological anomaly, almost a third of the women were aged 30–34. The second commonest age category was women aged 25–29, and the least common was those over 45. Of the 915 cases for termination due to a urological abnormality, less than 1% were women over 45, 4.2% were women under 19, 4.6% were those women aged between 40 and 44, 13.4% were women 20–24, 19.3% were aged 35–39, a quarter were those aged 25–29, and finally 32.3% were aged between 30 and 34.

### Ethnicity

The ethnicity of women undergoing termination of pregnancy for foetal urological abnormality is predominately those who class themselves as White (78.7%). A further 13.1% considered themselves Asian; 3.1% as Black; 1.2% as Mixed; 0.5% as Chinese and other; and the remaining 3.4% were not known or not stated.

### Marital Status

The vast majority of women included in this study were either married or in a civil partnership (46.8%) or single with a partner (31.1%). The remaining were either single without a partner (2.7%), single (partner not stated) (9.5%), separated/widowed/divorced (1.9%), or did not state an answer (8.0%).

### Obstetric History

Of all the women who underwent a termination for a foetal urological anomaly, 53.4% recorded having had one or more previous live births or stillbirths. This compares to an average of 55.0% of all abortions between 2015 and 2019 under any Ground. Over three-quarters (75.3%) of women having a termination for a foetal urological anomaly had not recorded a previous miscarriage or ectopic pregnancy, compared to an average of 80.0% of all abortions between 2015 and 2019 under any Ground. The remaining 226 cases (24.7%) had previously experienced one of the above outcomes. Only 13.9% of women having a termination for a foetal urological anomaly had previously had a legal termination of pregnancy, compared to an average of 38.6% of all abortions between 2015 and 2019 under any Ground. The remaining 788 cases (86.1%) had not previously had an abortion.

### Fetocide

**Fig. 7:**
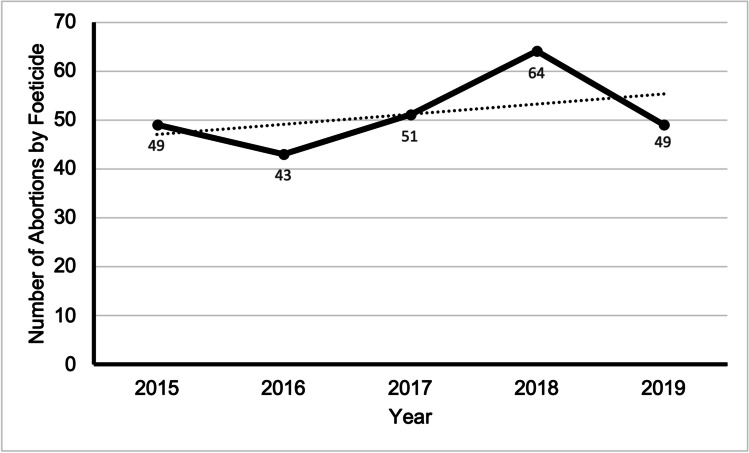
Graph to show the prevalence of abortion for urological abnormality by fetocide in residents and non-residents in England and Wales between 2015 and 2019. Data from the DHSC

For terminations over 21 + 6 weeks of gestation, the Royal College of Obstetricians and Gynaecologists (RCOG) advises fetocide because instances of recorded live birth and survival rise as gestation at birth increase from 22 weeks [7]. The recommended method for fetocide is intracardiac potassium chloride to guarantee foetal asystole. Between 2015 and 2019, the use of fetocide in terminations for urological abnormality has occurred in 28.0% (256 cases) of all cases (915) (Fig. 7). Of all Ground E terminations in 2019, 23% included fetocide.

## Discussion

This data suggests that the number of congenital malformations reported to the DHSC has broadly increased over the study period, with those done for urinary malformations rising. The abortions for malformations of the urinary system performed under Ground E rose from 4% (of the 45% of congenital malformations in 2015) to 5% (of the 53% of congenital malformations in 2019). Urological abnormality remains relatively rare and only accounts for 0.09% of the total number of abortions.

Our study was able to identify that the majority of cases were either isolated urinary anomalies or multiple anomalies of both urinary and extra-urinary systems (including chromosomal abnormalities). Of those cases with an isolated urinary anomaly, megacystis accounted for over one-fifth of the noted anomalies. Literature suggests that megacystis is more often associated with other urinary diagnoses, particularly posterior urethral valves (PUVs) rather than an isolate anomaly. One review found PUVs accompanied megacystis in 57.0% of cases [8]. Of cases reporting multiple urinary diagnoses, a third reported megacystis as one of the urinary codes recorded. Oligohydramnios was recorded in 44% of cases. In the ICD-10, this is classed as an extra-urinary anomaly though oligohydramnios is a direct result of abnormal urinary development. As amniotic fluid consists mainly of foetal urine after 16 weeks gestation, inability to effectively produce urine due to a congenital malformation will impact amniotic fluid volume. Loos et al. found that 37.0% of pregnancies affected by oligohydramnios were terminated following obstetric counselling, similar to the number of terminations of pregnancy where oligohydramnios was recorded in our study [9].

All the abortions associated with urological abnormalities in this study were TOPFA only. Though TOPFA is increasing in overall frequency and occurring at earlier gestations due to improved diagnostics, this data does not agree with our findings [10].@@@ Over half of the urological anomaly cases in the study period were terminated over 20 weeks, and no cases were terminated between 3 and 9 weeks. Urological malformations cannot be identified until after nine weeks, and therefore termination occurs significantly later in gestation, with some studies finding an average GA of diagnosis at 29.19 weeks [11]. Antenatal ultrasound is invariably the early screening modality for foetal anomaly scans and has proven to detect most malformations. However, lack of amniotic fluid impedes a thorough assessment of foetal anatomy, leading to inconclusive or diagnostically insufficient examinations. A recent study has demonstrated the benefit of foetal MRI, which on the other hand, is not significantly influenced by a lack of amniotic fluid or maternal obesity and therefore proves invaluable in establishing an accurate diagnosis and an earlier diagnosis of such urological anomalies [12].

In our study, medical termination for urological anomalies accounted for 90% of cases. Despite ROCG guidelines supporting a woman’s right to choose procedure modality, there is a disparity in service provision for TOPFA [13]. This difference may be due to preference, the desire for post-mortem examination, or limited availability of second-trimester surgical abortion within the NHS depending on geographical location as recognised by NICE. [14]

Results from our study show that termination for a foetal urological anomaly was the most common in the 30–34 age group, and the lowest rate of termination was for those women in the youngest (≤ 19) and oldest (40 +) age range. There are limited studies investigating the relationship between maternal age and CAKUT—Shanks et al. show that those above 34 years of age were less likely to be diagnosed with a foetal renal defect, after controlling for confounders, in comparison to women aged 34 and under; demonstrating older age is a protective factor in the absence of aneuploidy [15]. Parikh et al. investigated maternal age in relation to a specific diagnosis of renal agenesis and found that the effect of maternal age was variable; the odds of having a child with renal agenesis were increased in mothers under 18 years, yet maternal age older than 35 years proved to be a protective factor against having a child with the condition [16]. However, pregnancies in general occur most often in women aged 30–34 in the UK, and as such, these figures need to be interpreted with caution [17]. Despite being well established that older women are at an increased risk of certain anomalies like trisomies, there is limited data about maternal age and risk of urological abnormality and therefore TOPFA. More research relating age to the risk of urological anomaly and the physiological underpinnings is required.

The overwhelming majority of women who underwent TOPFA in our study were White. There is extremely limited published data on the ethnicity of women with a foetal urological diagnosis and terminating their pregnancy. Some studies suggest an increased prevalence of foetal anomalies in white women. Egbe et al. report that African Americans and Hispanics had lower risk, and Asians had a slightly elevated risk relative to Caucasians for genitourinary defects [18]. This broadly correlates with our data, though Asian women were still significantly underrepresented. Further research into the prevalence of urological malformation and termination in women of different ethnic origin could help identify any possible correlations.

Our study shows that nearly half of terminations that took place were in women who were either married or in a civil partnership. These outcomes differ from those of all abortions that took place within these 5 years, as the most common marital status to have a termination was in women who were single with a partner (53%), and 81% of women in 2019 who had an abortion of any kind were single (with or without a partner). Literature finds that abortion rates are usually the highest for single women [19]. Data to explain this stark difference in TOPFA and termination for any reason does not exist. This may be representative of change in cultural attitudes towards different family structures, including marriage.

Results from this study show that there was no statistically significant difference in women who had a previous pregnancy resulting in a live or stillbirth compared to those who had no previous pregnancies. This suggests that parity does not alter the likelihood of having a TOPFA due to a urinary system malformation. Nulliparity is usually associated with an increased risk of congenital anomaly, which contradicts our findings [20]. Moreover, having a previous miscarriage or ectopic pregnancy did not influence the prevalence of urological abnormality and TOPFA, as over 75% of women had no history of either outcome; likewise, a history of previous abortion under the Abortion Act did not result in an increased number of terminations. This is not reflected in the literature, as the risk of congenital anomalies is often higher in ‘high-risk’ pregnant women, including those with past miscarriages or ectopic pregnancies [21].

In 2019 there were 598 terminations under Ground E, performed over 21 + 6 weeks, thus qualifying for the recommended use of fetocide. In 91.8% of cases, fetocide was used, whereas, in the vast majority, fetocide accompanied a medical evacuation (489 points). Although the World Health Organisations recommends fetocide for TOP after 20 weeks gestation, there is no legal obligation in most European countries to perform this procedure in the context of TOPFA. Despite its use in the UK since the 1990s, it still raises concerns. Studies have examined the psychological experience of those involved, the technical aspects concerning the lethal substances used and route of administration, and ethical dilemmas [22]. Of particular relevance is a French study describing the use of fetocide in TOPFA; its results suggest that most TOPFAs during the second and third trimesters of pregnancy are practised after foetal anaesthesia and fetocide, where fetocide is almost always done after 22 weeks of gestation. Very few TOPFAs are organised without fetocide after 22 to 24 weeks [23].

Given that we were unable to compare the use of fetocide by gestational age in our study, it cannot be said whether the results from this study are in line with the literature and RCOG regulations. Our data shows that between 2015 and 2019, 28.0% of all abortions for urological anomaly involved the use of fetocide. It was established that 56.3% of TOPFA for urological abnormality took place at 20 weeks gestation and over. No data exists on the type of urological diagnosis and use of fetocide, and this study was also unable to look at individual diagnoses and the prevalence of fetocide. However, it would be rational to say that fatal conditions incompatible with life, such as BRA, would have a lower incidence of fetocide; conversely, those not lethal conditions such as hydronephrosis would have a more significant requirement for fetocide. This hypothesis is supported in literature on neural tube defects, where the universally fatal condition anencephaly had a minority of cases using fetocide, compared to almost 30% of spina bifida cases involving fetocide, depending on the seriousness and location of the lesion, is not lethal [24]. Earlier identification of abnormalities will allow women and their partners to arrive at an informed choice for TOPFA at an earlier GA. It may reduce the need for fetocide in the future.

## Summary

This was a first time study using national data from the DHSC archives to identify trends in termination of pregnancy for foetal urological abnormalities across England and Wales over 5 years. Overall, this study shows that termination cases for a urinary system anomaly have remained similar over the study period. The mean gestation for TOPFA associated with urological anomaly was 20 weeks and above, which is likely due to the detection of urological abnormalities during the second trimester. Such terminations were most commonly performed in White mothers aged 30–34 years. Notably, the decision to undergo a TOPFA is likely multifactorial, and this study did not directly compare parameters within patient demographics.

A medical method of abortion was the most common, possibly due to a recognised lack of choice of surgical techniques within an NHS setting, particularly regarding later surgical procedures [14].

Of all abortions mentioning urological anomaly, 28.0% involved the use of fetocide, and this figure may appear small given that over half of the terminations took place at 20 weeks and over when the RCOG recommends fetocide. There was no association between a previously adverse obstetric history, notably past miscarriage, ectopic pregnancy, or abortion, and between multiparous and nulliparous women. Finally, most cases were isolated urinary anomalies or multiple anomalies, including urinary and extra-urinary systems, where nearly 95.0% of cases were recorded as either.

## Limitations

This study is limited by its retrospective nature, relatively small sample size, and short study duration. Data were extracted from the DHSC in an aggregated format rather than patient-level data. Confidentiality concerns limited raw data extraction, as identifiable information such as gestation or maternal age was presented in ranges. Furthermore, no data was provided on each specific diagnosis/ICD10 code in any category, and neither was data provided directly comparing one type to another, i.e., maternal age (at time of termination) by gestation of the foetus; this would have allowed for a much more thorough evaluation of trends. Further research is required to analyse these trends to a greater extent.

## Further Research

Congenital anomalies of the kidney and urinary tract encompass a disparate group of diseases, requiring a multidisciplinary approach for accurate prenatal diagnosis and thus improved prognoses. Our study shows that a significant number of cases identified are terminated nationally. This highlights the need for early diagnosis and the development of robust literature on the prognosis of specific abnormalities. Such factors are the prerequisites for making informed decisions on the termination of pregnancy for foetal anomalies. Further research is required to identify in more detail which maternal characteristics are associated with severity and termination and possible external risk factors for foetal urological malformation, as the literature is limited and inconclusive.

## Conclusions

Foetal urological abnormality is a complex issue affecting a significant minority of pregnant women. When severe abnormalities are detected by prenatal diagnosis, most women choose to terminate the pregnancy. Yet, despite the wide availability of antenatal screening, termination of pregnancy rates for foetal urological abnormality have remained linear over the last 5 years. This study highlights the need for further research over a more extended period to establish more substantial trends between maternal characteristics and developments of urological anomalies and factors that may influence a woman to proceed or not to proceed with termination.

## Data Availability

Data subject to third-party restrictions. The data that support the findings of this study are available from The Department of Health and Social Care. Restrictions apply to the availability of these data, which were used under license for this study. Data are available from the authors with the permission of The Department of Health and Social Care.
